# Comprehensive analysis of the diagnostic and therapeutic value, immune infiltration, and drug treatment mechanisms of GTSE1 in lung adenocarcinoma

**DOI:** 10.3389/fmed.2024.1433601

**Published:** 2024-11-19

**Authors:** Guanqiang Yan, Jingxiao Li, Xiang Gao, Jun Liu, Guiyu Feng, Yue Li, Huafu Zhou

**Affiliations:** ^1^Department of Cardio-Thoracic Surgery, The First Affiliated Hospital of Guangxi Medical University, Nanning, China; ^2^Department of Guangxi Medical University, Nanning, China

**Keywords:** GTSE1, lung adenocarcinoma, bioinformatics, immune infiltration, targeted therapy

## Abstract

**Objective:**

The aim of this investigation was to assess the diagnostic and therapeutic efficacy of G2 and S-phase expressed 1 (GTSE1) in lung adenocarcinoma (LUAD), while examining its impact on immune infiltration and drug treatment mechanisms.

**Methods:**

This research involved examining the expression patterns and diagnostic accuracy of GTSE1 in LUAD using various databases and clinical samples. The databases utilized included Gene Expression Omnibus (GEO), Clinical Proteomic Tumor Analysis Consortium (CPTAC), and The Cancer Genome Atlas (TCGA). Both gene expression and protein levels were analyzed. Subsequently, the prognostic ability of GTSE1 was evaluated based on clinical follow-up data using methods such as using univariate, multivariate, and prognostic meta-analysis. Additionally, potential mechanisms of action of GTSE1 were explored through enrichment analysis. Furthermore, the correlation between GTSE1 expression and the tumor microenvironment, immune cell infiltration, and immune checkpoints was assessed using ESTIMATE and CIBERSORT algorithms. The effectiveness of chemotherapy and targeted therapy was predicted using the “pRophetic” R package, which analyzed gene expression data.

**Results:**

Analysis of GEO data, CPTAC data, TCGA data, and clinical samples revealed increased levels of GTSE1 in LUAD tissues. Enhanced GTSE1 expression demonstrated excellent diagnostic accuracy and served as a significant prognostic indicator for LUAD patients. GTSE1 expression emerged as an independent predictive factor in both univariate and multivariate Cox regression analyses. Furthermore, functional enrichment analysis suggested a potential association between GTSE1 and the cell cycle, p53 signaling pathway, as well as ubiquitin-mediated protein degradation. High expression of GTSE1 was associated with increased immune cell infiltration and heightened sensitivity to a specific type of chemotherapy and targeted drugs.

**Conclusion:**

Increased expression of GTSE1 in patients with LUAD showed significant diagnostic and prognostic significance. It was also associated with increased immune infiltration and an unfavorable response to targeted medication.

## Introduction

Lung cancer (LC) is the most prevalent and deadliest cancer type in China. Recent research reports have indicated that LC ranks second in incidence and first in mortality among malignant tumors worldwide (1, 2). Lung adenocarcinoma (LUAD), the most commonly diagnosed subtype of LC, accounts for approximately 40% of LC cases based on pathological classification (3). Despite advancements in diagnostic and treatment techniques, the combination of surgery, radiotherapy, and chemotherapy has achieved a 5-year survival rate of over 20% for LUAD patients (4). However, an increasing number of patients are being diagnosed at advanced stages of cancer, compromising the efficacy of conventional treatment methods (5). In recent years, scientists have suggested biomarkers, including exosomes, proteins, and microRNAs, for the early detection and treatment of LUAD. However, limited adoption of these biomarkers can be attributed to stringent application conditions and unsatisfactory diagnostic and therapeutic outcomes. Therefore, there is a crucial need to explore effective clinical screening and treatment biomarkers for non-small cell lung cancer to significantly reduce patient mortality rates and enhance their overall quality of life.

G2 and S-phase expressed 1 (GTSE1), located at 22q13.31, is believed to be activated by p53, leading to the inhibition of tumor cell apoptosis (6). GTSE1 has been implicated in the control of tumor initiation and progression in various malignancies, including breast cancer, prostate cancer, and colon cancer (7–9). Previous research has indicated GTSE1’s regulatory role in LC. Initially, researchers conducted an analysis of public datasets and developed a risk prediction model, identifying several potential LC risk genes, including GTSE1 (10). Following extensive research, a group of scientists led by Zhang et al. and Wang et al. discovered that increased levels of GTSE1 were crucial in promoting the dissemination and movement of LC cells. This effect was achieved by activating the AKT/mTOR and ERK/MAPK signaling pathways. Intriguingly, when GTSE1 expression was suppressed, LC cells exhibited a substantial reduction in their ability to invade and metastasize. Additionally, they displayed heightened responsiveness to radiotherapy, suggesting GTSE1 as a potential therapeutic target in LC treatment (11, 12). However, conflicting findings exist in the literature regarding the association between GTSE1 expression and LC progression. While prior research has reported a clear increase in GTSE1 expression in LC tissues, its differential expression has not demonstrated significant influence on patients’ clinical features or survival rate (13). These inconclusive conclusions necessitate further investigation to clarify the role of differentially expressed GTSE1 in LC cell development. Moreover, the regulatory effects and mechanisms of the same biomarker may differ due to variations in pathological types, which could contribute to the contrasting conclusions. To date, no studies have reported the role and potential mechanisms of GTSE1 in various subtypes of LC. Furthermore, the regulatory role and mechanisms of GTSE1 in LUAD remain unclear. Therefore, it is crucial to investigate the expression levels and functional mechanisms of GTSE1 in LUAD tissues using a large number of samples to clarify the potential clinical applicability of GTSE1.

In this study, the gene expression profiles from public databases and samples obtained from our hospital were analyzed. The aim was to examine GTSE1 expression in LUAD tissues and assess its diagnostic potential for the disease.

## Methods

### Data acquisition

The information used in this research was collected from various sources. Clinical data and RNA sequencing gene expression data for LUAD were gathered from three major databases: the Gene Expression Omnibus (GEO), the Clinical Proteomic Tumor Analysis Consortium (CPTAC), and The Cancer Genome Atlas (TCGA). These databases served as valuable resources for researchers in the field. GEO, accessible at https://www.ncbi.nlm.nih.gov/geo/, provided comprehensive gene expression data from various organisms. CPTAC, available at https://proteomics.cancer.gov/programs/cptac/, focused on proteomic analysis of tumor samples. Lastly, TCGA, accessible at https://www.portal.gdc.cancer.gov/, provided a wealth of information on genomic alterations in cancer. By utilizing these databases, researchers were able to obtain the necessary data for this study. Additionally, specimens obtained from individuals diagnosed with LUAD at The First Affiliated Hospital of Guangxi Medical University were incorporated. The selected specimens needed to possess GTSE1 sequencing information as well as comprehensive clinical data pertaining to LUAD patients, including factors such as age, gender, pathological TNM staging, and survival outcomes for microarray analysis, a search was conducted in the GEO database using the following search criteria: (pulmonary, respiratory, lung, bronchial, bronchiole, alveolar, lung cell, respiratory tract) combined with (carcinoma, cancer, tumor, malignant tumor, adenocarcinoma). The cut-off for inclusion was May 2023. The GEO datasets selected had to meet the following criteria: (1) complete GTSE1 expression data; (2) studies that included both tumor and control groups; (3) each group consisted of at least 20 samples. The “sva” R package was used to remove batch effects from GSE microarray data obtained from the same platform, followed by standardization using the “limma” R package. The processed GSE microarray data were then integrated into a GPL dataset for analysis. Additionally, a study was conducted at the First Affiliated Hospital of Guangxi Medical University, wherein 15 samples of tumor and adjacent tissue were collected from patients diagnosed with LUAD. It was worth noting that none of these 15 patients received neoadjuvant therapy before undergoing surgery. The Ethics Committee of the First Affiliated Hospital of Guangxi Medical University provided approval for this study.

### Meta-analysis

Stata 12.0 software was used to conduct a meta-analysis on microarray datasets retrieved from the GEO database. To evaluate heterogeneity among the studies, the *I*^2^ statistic and Cochran’s *Q* test were employed. Studies with *p* > 0.05 and *I*^2^ < 50% were categorized as having consistent outcomes. Higher heterogeneity was detected if the *p*-value was less than 0.05 or if *I*^2^ exceeded 50%. In cases of higher heterogeneity, a random-effects model was used to estimate the combined effect size, while a fixed-effects model was used otherwise. In the context of diagnostic meta-analysis, the discrimination threshold among diagnostic studies was established by evaluating the receiver operating characteristic (ROC) curve and Spearman’s rank correlation coefficient. Summary statistics were then calculated for sensitivity and specificity, along with their respective 95% confidence intervals. The calculation included determining the area under the curve (AUC) of the summary receiver operating characteristic (SROC) curve. Visual representations of the data were created through forest plots and SROC plots. Additionally, a reliability assessment was conducted by systematically excluding each study to perform a sensitivity analysis and evaluate the findings. Recombination of effect sizes was conducted to assess stability. The assessment of publication bias in the aforementioned studies was carried out using Deek’s funnel plot. In the meta-analysis of prognosis, we combined the hazard ratio (HR) and the corresponding 95% confidence interval (CI) to assess the predictive importance of GTSE1 in individuals diagnosed with LUAD. The low-expression group of GTSE1 was taken as the baseline for the examination. It was observed that an HR value surpassing 1 denoted a considerably poorer prognosis in the high-expression group of GTSE1. Finally, Begg’s and Egger’s tests were applied to assess the potential for publication bias, with *p*-values below 0.05 considered indicative of noteworthy statistical disparities.

### Quantitative real-time PCR analysis

After operation, the LUAD tissue was cut off and placed in a freezing centrifuge tube, frozen in liquid nitrogen and stored at −80°C. Frozen lung tissue was utilized to isolate RNA completely using TRIzol reagent from Vazyme, China. Then, cDNA was synthesized by reverse transcribing 2 μg of RNA using a kit from Vazyme, China. The primers employed were GTSE1 forward: 5′-CAGGGGACGTGAACATGGATG-3′; GTSE1 reverse: 5’-ATGTCCAAAGGGTCCGAAGAA-3′; *β*-actin forward: 5′-TCACCCACACTGTGCCCATCTACGA-3′; β-actin reverse: 5′-CAGCGGAACCGCTCATTGCCAATGG-3′. Quantitative real-time PCR was conducted using SYBR Green. The quantification of changes in expression was determined using the $2^{-\Delta \Delta C_{T}}$ approach and compared to the relative expression level of β-actin. Each qRT-PCR reaction was carried out in triplicate.

### Western blotting

When extracting proteins from tissues and quantifying them, it was crucial to adhere to the guidelines provided by the manufacturer. The proteins were separated using SDS-PAGE and transferred onto a PVDF membrane. A GTSE1 antibody (rabbit polyclonal antibody, Proteintech, Catalog No. 21319-1-AP) was applied to a probe membrane that had been sealed with 5% skim milk for 1 h and incubated at 4 degrees celsius overnight. Beta Actin Monoclonal antibody (mouse IgM isotype, Proteintech, Catalog No. 60008-1-Ig) served as the internal control. The Bio-rad Gel Doc system was employed for visualization and analysis of the bands.

### Gene enrichment analysis and GSEA analysis

The TCGA LUAD samples were divided into two categories according to the median expression level of GTSE1. Genes showing differential expression were selected using the criteria of FDR <0.05 and |log2FC| >1. The “clusterProfiler” R package was used to annotate the differentially expressed genes (DEGs) using Gene Ontology (GO) to explore their biological functions. Additionally, GSEA (version 4.2.3) was utilized to investigate the enrichment of distinct gene sets between the two groups.

### Analysis of LUAD immune microenvironment characteristics and drug susceptibility analysis

The immune score, stromal score, and estimate score for each sample in the TCGA dataset were calculated using the ESTIMATE algorithm. The infiltration of 22 distinct immune cell types in each LUAD sample was evaluated using the CIBERSORT algorithm, as these cells play a critical role in the tumor immune microenvironment. Further exploration was conducted to examine the correlation between the scores and the expression levels of GTSE1. The R programming language was used to perform single-sample gene set enrichment analysis (ssGSEA) using the GSVA package. This facilitated the evaluation of the abundance of 13 immune markers within the tumor immune microenvironment. Additionally, the relationship between GTSE1 and immune checkpoint genes was investigated, considering its potential influence on the effectiveness of therapeutic drugs. Moreover, IC_50_ values were determined using the “pRRophetic” R package. The focus was on comparing the efficacy of commonly prescribed medications for LUAD among patients with varying levels of disease severity.

### Statistical analysis

Statistical analysis was performed using R (version 4.2.0) and SPSS (version 25.0) in this investigation. *T*-tests or Wilcoxon rank-sum tests were used to evaluate the levels of GTSE1 in tumor tissues and normal tissues. The relationship between GTSE1 expression and clinical pathological features was assessed through Kruskal–Wallis tests, Wilcoxon signed-rank tests, and logistic regression analysis. COX risk regression analysis was employed to determine the prognostic value of GTSE1 expression. The significance of Kaplan–Meier survival analysis was assessed using the log-rank test.

## Results

### GTSE1 was up-regulated in LUAD patients

**Figure 1:**
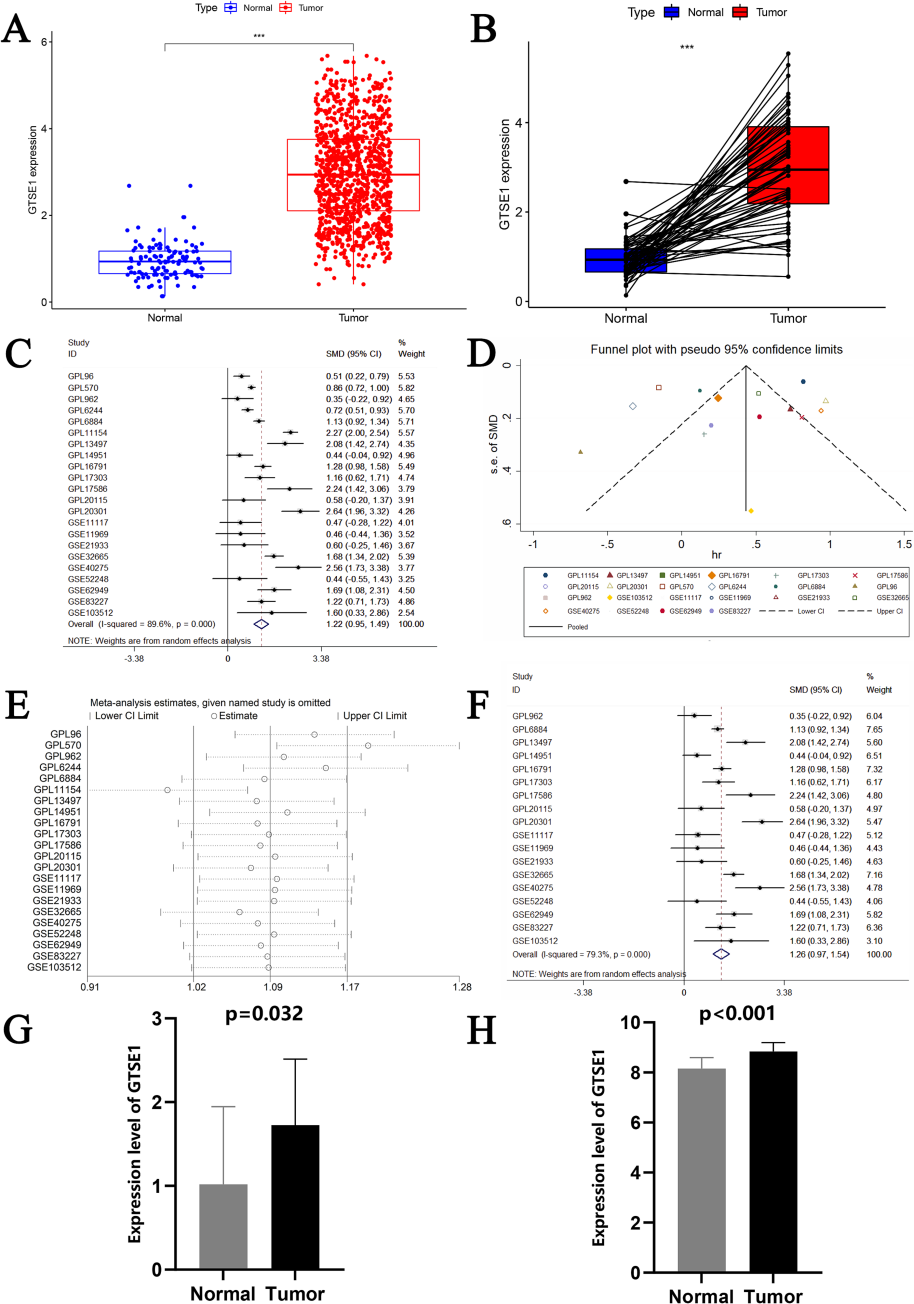
The expression level of GTSE1 in multiple validation datasets and meta-analysis of GEO datasets. (A,B) GTSE1 mRNA expression level in TCGA-LUAD cohort, based on single sample *t*-test and paired-samples *t*-test. (C) Forest plot of included 22 studies. (D) Funnel plot. (E) Forest plot of sensitivity analysis based on deleting literature one by one. (F) Forest plot of finally included 18 studies. (G) GTSE1 mRNA expression level in LUAD tissue. (H) GTSE1 mRNA expression level in clinical samples.

This study obtained data from 539 LUAD tissues and 59 adjacent non-cancerous tissues collected by TCGA. Differential analysis was conducted to determine the expression level of GTSE1 in lung adenocarcinoma tissues. The results showed a significantly higher expression level of the GTSE1 gene in LUAD tissues compared to normal lung tissues (Figure 1A). This conclusion was further confirmed by pairing the TCGA sample cohort (Figure 1B). We collected 22 datasets from GEO to validate these findings. Heterogeneity analysis showed significant heterogeneity among the 22 included studies (*I*^2^ = 89.6%, *p* < 0.001). Meta-analysis using a random effects model showed a pooled standardized mean difference (SMD) value of 1.22 (Figure 1C), indicating a higher expression of GTSE1 mRNA in cancer tissues compared to normal tissues and adjacent non-cancerous tissues. Sensitivity analysis demonstrated that the meta-analysis stability improved when the random effects model was utilized.

**Figure 2:**
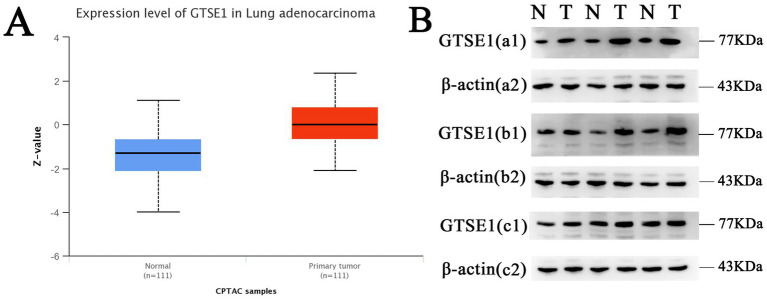
The protein expression level of GTSE1. (A) GTSE1 protein expression level in CPTAC dataset. (B) GTSE1 protein expression level in clinical samples; “N” stands for “Normal”, “T” stands for “Tumor”.

The datasets GPL96, GPL570, GPL6244, and GPL11154 exhibited higher heterogeneity (Figure 1D) and more bias in the publication bias analysis (Figure 1E). Exclusion of these datasets decreased heterogeneity, with a combined standardized mean difference (SMD) of 1.26 (Figure 1F). RT-PCR analysis of GTSE1 expression levels in LUAD tissue showed significant up-regulation compared to normal lung tissue (Figure 1G, *p* < 0.05). Additionally, analysis of the GSE32665 dataset demonstrated lower expression levels of GTSE1 in normal tissue compared to LUAD tissue (Figure 1H). This finding suggests a potential role of GTSE1 in LUAD. The upregulation of GTSE1 protein in cancer samples was further confirmed in 111 pairs of LUAD samples obtained from the CPTAC database (Figure 2A). Protein immunoblotting was utilized to determine GTSE1 protein expression levels. It was observed that the expression level of GTSE1 protein was relatively higher in LUAD tissues, providing additional support for our conclusion (Figure 2B).

### Diagnostic value of GTSE1 in LUAD patients

**Figure 3:**
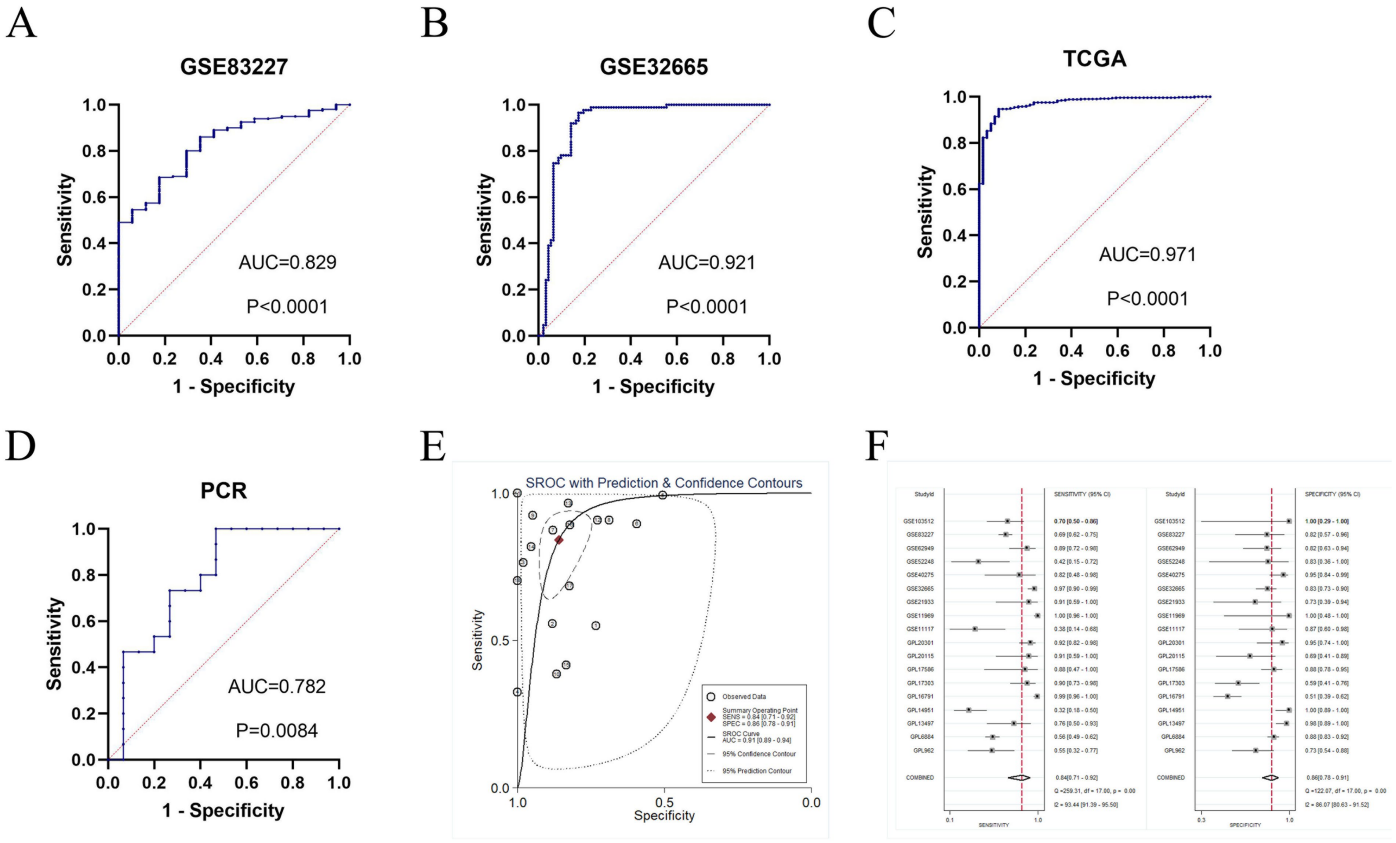
The diagnosis value of GTSE1 in LUAD patients. (A–D) ROC curves of GSE83227, GSE32665, TCGA and clinical samples cohort. (E) sROC curve of included 18 studies (AUC = 0.91). (F) Forest plot for sensitivity and specificity.

The diagnostic potential of GTSE1 for LUAD was assessed through a diagnostic meta-analysis. Computational results from GSE83227, GSE32665, TCGA, and the experimental cohort demonstrated that GTSE1 had a substantial diagnostic potential (Figures 3A–D, with AUC values of 0.829, 0.921, 0.971, and 0.782 respectively). Among the 18 studies included, the area under the fitted SROC curve was 0.91 (95% CI 0.89–0.94) (Figure 3E). The comprehensive effect size analysis yielded a diagnostic sensitivity of 0.84 (95% CI 0.71–0.92), specificity of 0.86 (95% CI 0.78–0.91), positive likelihood ratio (PLR) of 6.0 (95% CI 3.9–9.1), negative likelihood ratio (NLR) of 0.18 (95% CI 0.10–0.35), and diagnostic odds ratio of 32 (95% CI 15–68) (Figure 3F and Table 1). These results unequivocally demonstrated the high diagnostic value of GTSE1 for LUAD.

**Table 1:** Summary results of diagnostic meta-analysis.

Parameter	Estimate	95% CI
Sensitivity	0.84	[0.71, 0.92]
Specificity	0.86	[0.78, 0.91]
Positive likelihood ratio	6.0	[3.9, 9.1]
Negative likelihood ratio	0.18	[0.10, 0.35]
Diagnostic odds ratio	32	[15, 68]

### GTSE1 expression was associated with clinicopathological characteristic and survival outcomes in LUAD patient

To elucidate the role of GTSE1 in LUAD progression, we analyzed the relationship between its expression and clinical follow-up data in LUAD patients. Our findings revealed a significant correlation between GTSE1 expression and age, gender, T-stage, and M-stage in the TCGA group (*p* < 0.05) (Supplementary Figures S1A–F). Notably, GTSE1 expression was markedly elevated in LUAD patients at the M0 stage (Supplementary Figure S1D).

**Figure 4:**
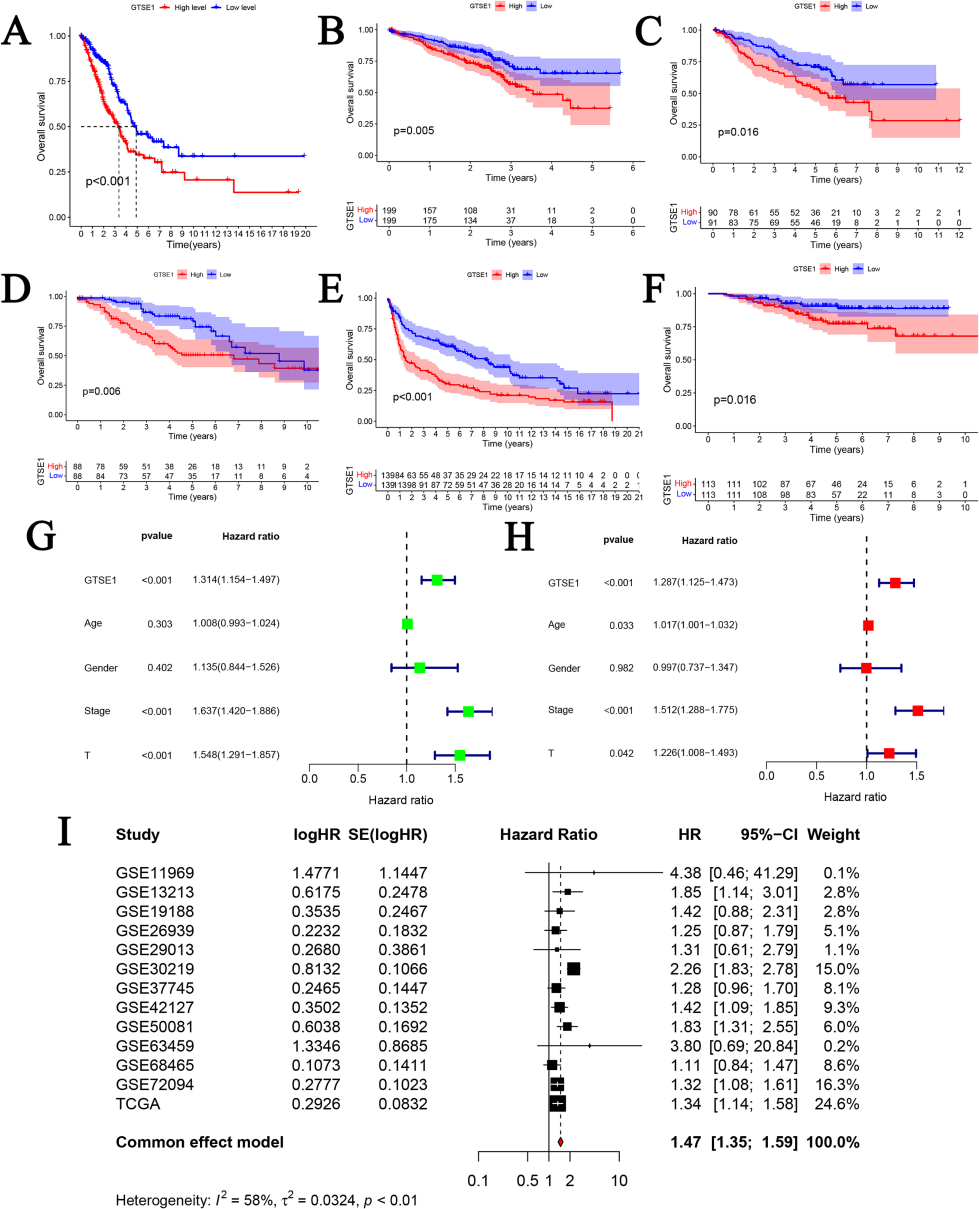
The prognosis value of GTSE1 in LUAD patients. (A–F) Kaplan–Meier OS curves in TCGA-LUAD, GSE72094, GSE50081, GSE42127, GSE30219 and GSE31210 cohort. (G) Univariate cox regression analysis. (H) Multivariate cox regression analysis. (I) Prognostic meta-analysis of included 13 studies (combined HR = 1.47).

To assess the predictive capacity of GTSE1, we conducted a survival analysis using the median value of GTSE1 expression to compare the prognosis between the high and low expression groups. In the TCGA-LUAD dataset, patients with lower levels of GTSE1 expression showed significantly improved overall survival compared to those with higher levels of GTSE1 expression (Figure 4A). This observation was consistently seen in the GSE72094, GSE50081, GSE42127, GSE30219, and GSE31210 datasets, indicating that individuals with higher GTSE1 expression experienced shorter overall survival (Figures 4B–F). We examined the relationship between GTSE1 expression level (HR = 1.314, *p* < 0.001), T stage (HR = 1.548, p < 0.001), pathological stage (HR = 1.637, *p* < 0.001), and overall survival (OS) in patients with LUAD using univariate regression in the TCGA database, and significant associations were found (Figure 4G). Furthermore, multivariate Cox regression analysis demonstrated that GTSE1 expression remained a significant prognostic factor independent of other risk factors (HR = 1.287, *p* < 0.001) (Figure 4H). To further validate these findings, we performed a comprehensive analysis by combining prognostic data from 13 studies in the GEO and TCGA databases using a random-effects model. The results consistently indicated that increased GTSE1 expression correlated with worse overall survival in individuals diagnosed with LUAD (HR = 1.47, 95% CI 1.35–1.59) (Figure 4I).

### Potential mechanism of GTSE1 in LUAD

**Figure 5:**
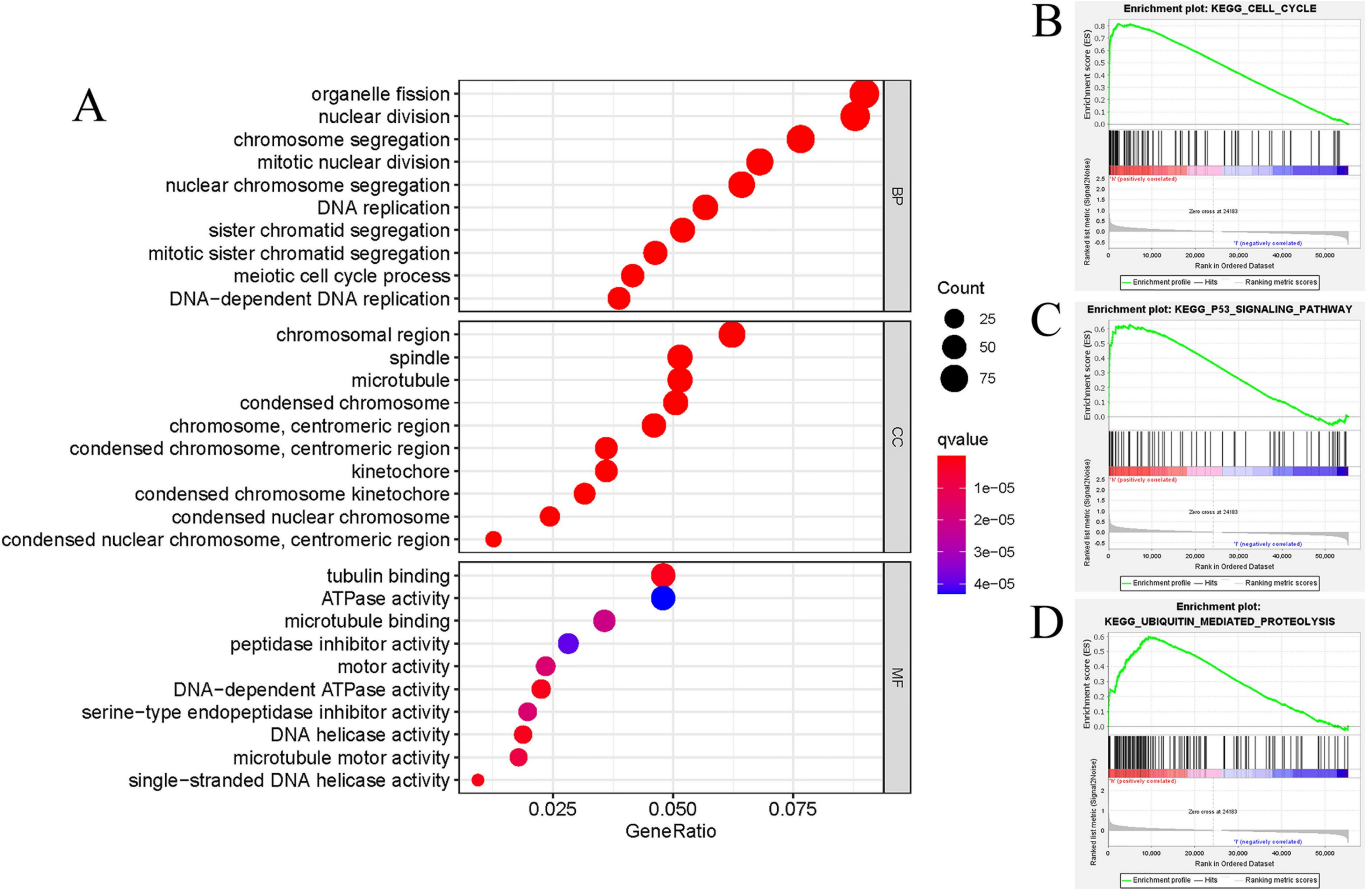
Functional annotation of DEGs in LUAD patients with distinct GTSE1 levels. (A) GO enrichment analysis of DEGs in GTSE1 high expression group. (B–D) Representative gene set enrichment analysis between GTSE1 high and low expression groups.

The study revealed a potential association between GTSE1 and the development and progression of LUAD. To investigate the biological activity of GTSE1, we performed Gene Ontology (GO) enrichment analysis using differentially expressed genes (DEGs) between the high-expression and low-expression groups. The results showed significant enrichment of DEGs in immune-related biological processes in the TCGA cohort, including humoral immune response, antibacterial humoral immune response, bone marrow leukocyte migration, neutrophil chemotaxis, and migration (Figure 5A). Subsequently, we utilized the gene set enrichment analysis (GSEA) method to identify differentially enriched gene sets between the up-regulated and down-regulated GTSE1 groups. Twenty gene sets were found to be enriched in the up-regulated GTSE1 group, including the cell cycle, p53 signaling pathway, and ubiquitin-mediated protein degradation (Table 2 and Figures 5B–D). These findings suggest a close association between GTSE1 and cancer occurrence, with a potential role in immune regulation within the tumor microenvironment.

**Table 2:** Gene sets enriched in GTSE1 high expression group.

Gene set details	ES	NES	NOM *p*-val	FDR *q*-val
KEGG_OOCYTE_MEIOSIS	0.65	2.56	<0.001	<0.001
KEGG_SPLICEOSOME	0.68	2.56	<0.001	<0.001
KEGG_UBIQUITIN_MEDIATED_PROTEOLYSIS	0.60	2.55	<0.001	<0.001
KEGG_CELL_CYCLE	0.81	2.54	<0.001	<0.001
KEGG_PROGESTERONE_MEDIATED_OOCYTE_MATURATION	0.65	2.43	<0.001	<0.001
KEGG_RNA_DEGRADATION	0.64	2.42	<0.001	<0.001
KEGG_NUCLEOTIDE_EXCISION_REPAIR	0.72	2.33	<0.001	<0.001
KEGG_BASAL_TRANSCRIPTION_FACTORS	0.67	2.29	<0.001	<0.001
KEGG_PYRIMIDINE_METABOLISM	0.62	2.27	<0.001	<0.001
KEGG_HOMOLOGOUS_RECOMBINATION	0.88	2.27	<0.001	0.001
KEGG_P53_SIGNALING_PATHWAY	0.63	2.24	<0.001	0.001
KEGG_MISMATCH_REPAIR	0.89	2.19	<0.001	0.002
KEGG_CYSTEINE_AND_METHIONINE_METABOLISM	0.62	2.18	<0.001	0.002
KEGG_BASE_EXCISION_REPAIR	0.73	2.11	<0.001	0.004
KEGG_PROTEASOME	0.72	2.06	0.002	0.008
KEGG_DNA_REPLICATION	0.90	2.05	<0.001	0.008
KEGG_PATHOGENIC_ESCHERICHIA_COLI_INFECTION	0.53	1.99	<0.001	0.014
KEGG_N_GLYCAN_BIOSYNTHESIS	0.52	1.99	0.002	0.014
KEGG_PANCREATIC_CANCER	0.54	1.97	0.008	0.016
KEGG_SMALL_CELL_LUNG_CANCER	0.58	1.97	<0.001	0.016

### Analysis of tumor immune microenvironment in various expression groups

**Figure 6:**
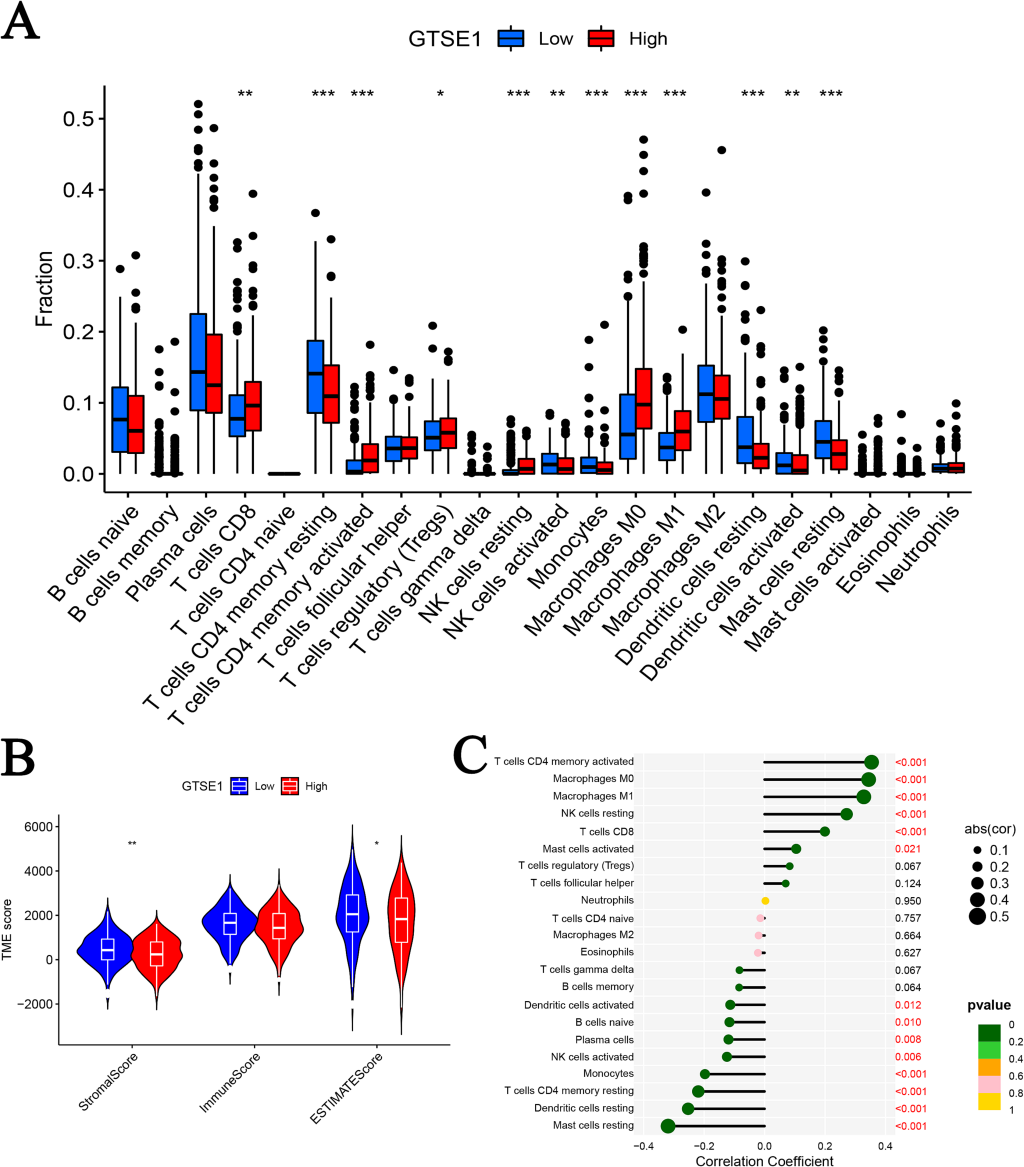
GTSE1 expression and tumor immune microenvironment. (A) Infiltration of 22 immune cells between two groups. (B) TME scores between GTSE1 high and low groups. (C) Correlation between 22 immune cells and GTSE1 (^*^p < 0.05, ^**^p < 0.01, and ^***^p < 0.001).

Subsequently, we conducted a detailed investigation into the correlation between GTSE1 expression and the tumor immune microenvironment. Our analysis of the TCGA dataset revealed that patients with increased GTSE1 expression displayed significant elevations in stromal score, immune score, and estimate score (Figure 6B). Using the CIBERSORT algorithm, we further examined the distribution of 22 immune cell types and compared their presence in the groups with upregulated and downregulated GTSE1 expression. Notable disparities in immune cell distribution were observed between these two groups. Specifically, CD8^+^ T cells, activated memory CD4^+^ T cells, resting NK cells, M0 macrophages, and M1 macrophages exhibited higher infiltration in the group with upregulated GTSE1 expression, while resting memory CD4^+^ T cells, activated NK cells, monocytes, resting dendritic cells, and resting mast cells showed poorer infiltration (Figure 6A). Moreover, we conducted Spearman’s correlation analysis to assess the association between GTSE1 expression levels and immune cell infiltration scores. The analysis revealed significant positive correlations between GTSE1 expression levels and the infiltration scores of various immune cell types, including activated memory CD4^+^ T cells, M0 macrophages, M1 macrophages, resting NK cells, CD8^+^ T cells, and activated mast cells (Figure 6C, *p* < 0.05). Conversely, resting mast cells, resting dendritic cells, resting memory CD4^+^ T cells, monocytes, activated NK cells, plasma cells, naive B cells, and activated dendritic cells demonstrated significant negative correlations (Figure 6C, *p* < 0.05).

**Figure 7:**
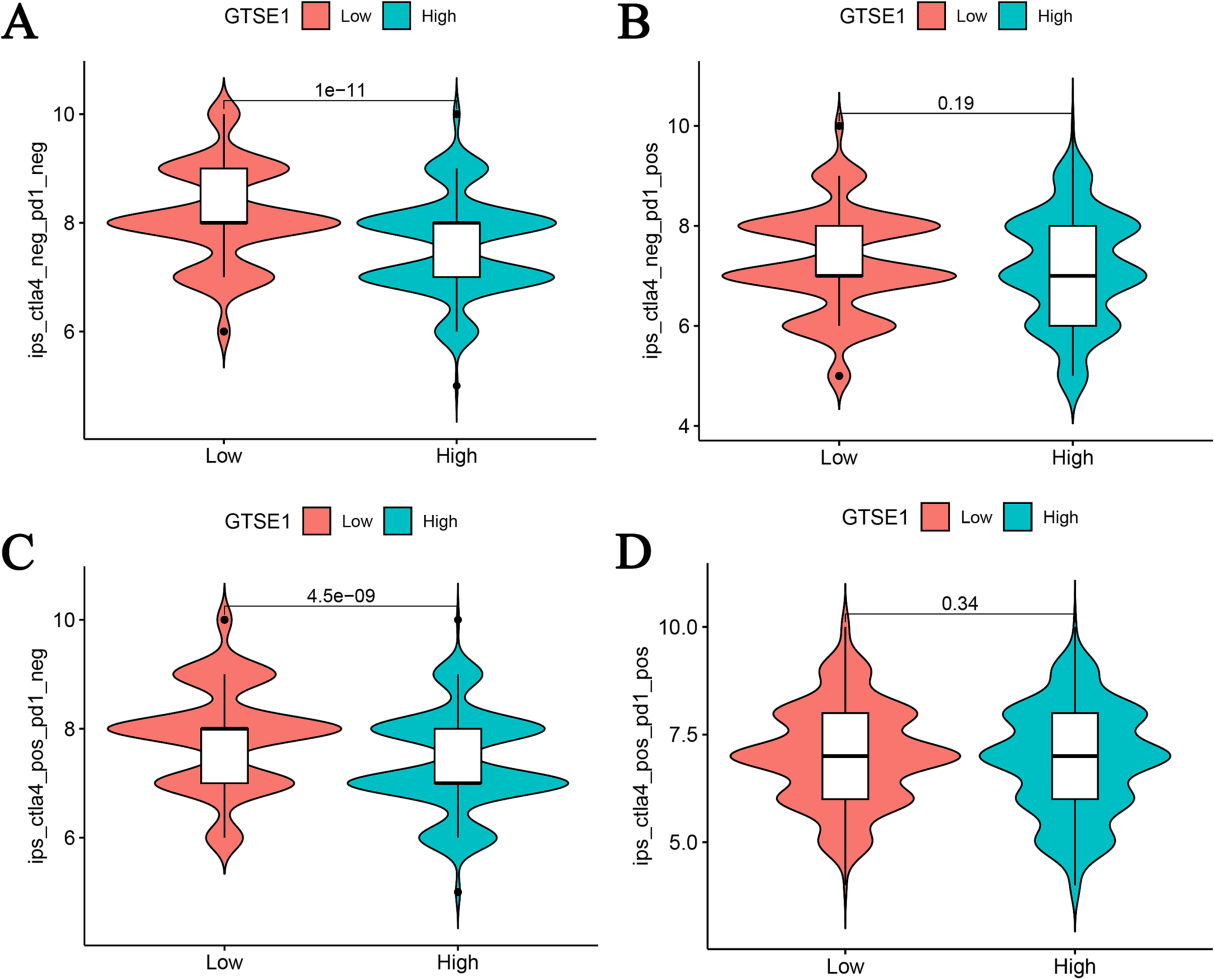
IPS analysis between high and low GTSE1 expression groups. (A) CTLA4^−^ and PD1^−^, (B) CTLA4^−^ and PD1^+^, (C) CTLA4^+^ and PD1^−^, (D) CTLA4^+^ and PD1^+^.

Furthermore, we utilized the CIBERSORT algorithm to perform correlation analysis in order to elucidate the connection between the abundance of immune cells and CRG risk scores. Our results unveiled a positive correlation between CRG risk scores and M0 macrophages, M1 macrophages, activated mast cells, resting NK cells, activated CD4 memory T cells, and CD8 T cells. Conversely, CRG risk scores demonstrated a negative correlation with naive B cells, activated dendritic cells, resting dendritic cells, resting mast cells, monocytes, activated NK cells, plasma cells, and resting CD4 memory T cells (Supplementary Figures S2A–N). Considering the significant changes in immune checkpoint expression levels between the upregulated and downregulated GTSE1 groups (Supplementary Figure S3), we further investigated the potential predictive value of GTSE1 on the efficacy of immunotherapy. In the “CTLA4^+^ PD1^−^” group, the low expression group of GTSE1 exhibited a higher mean immune predictive score (IPS). However, there was no significant difference in the average IPS between the upregulated and downregulated GTSE1 groups in the “CTLA4^−^ PD1^+^” group (Figures 7A–D). These findings suggest that individuals with low GTSE1 expression levels may benefit more from treatment with CTLA4^+^.

### The role of GTSE1 expression level in predicting therapeutic efficacy

**Figure 8:**
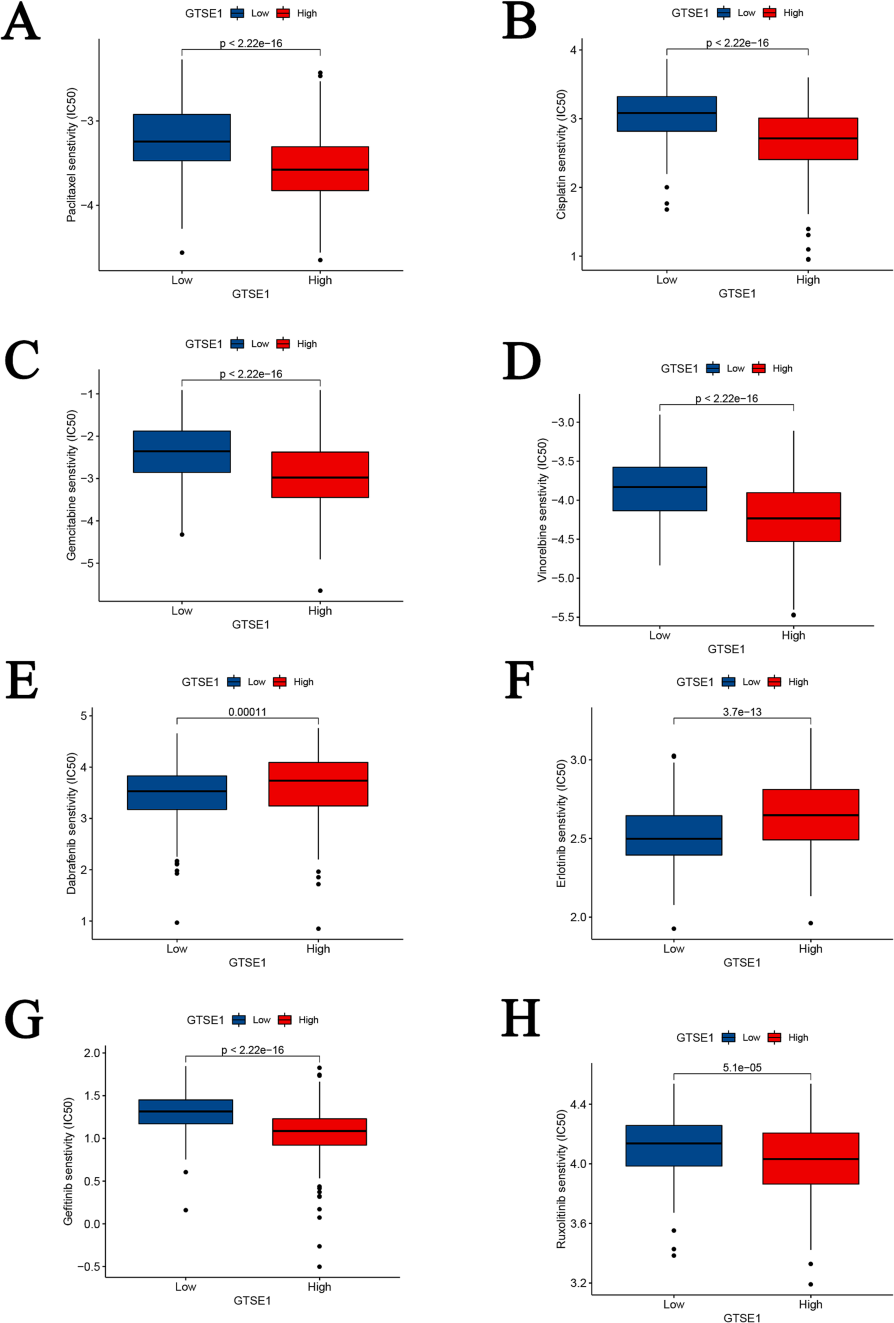
Drug sensitivity analysis between GTSE1 high and low groups. (A–D) Chemotherapeutic drugs. (E–H) Targeted drugs.

To determine the IC_50_ values of chemotherapy and targeted drugs on LUAD tumor cells, we utilized the “pRRhetic” package in R software. The results indicated that in the high expression group of GTSE1, the IC_50_ values of cytotoxic chemotherapy drugs (paclitaxel, cisplatin, gemcitabine, and irinotecan) were lower compared to the low expression group (Figures 8A–D), suggesting a higher sensitivity to these drugs in the high expression group. Additionally, the targeted drugs dabrafenib and erlotinib exhibited substantially higher IC_50_ values in the GTSE1 high expression group, while gefitinib and ruxolitinib displayed the opposite trend (Figures 8E–H), indicating lower sensitivity of dabrafenib and erlotinib in the high expression group, whereas gefitinib and ruxolitinib exhibited higher sensitivity.

## Discussion

Previous studies have shown that GTSE1 is upregulated in lung cancer (LC) and is considered a potential risk gene for LC development (10, 14, 15). However, LC encompasses various subtypes, such as LUAD, squamous cell carcinoma, and small cell lung cancer, and the molecular mechanisms and pathways driving their development may not consistently align, leading to conflicting research findings (13). Therefore, the objective of this research was to gather and assess LUAD samples from public databases and a hospital affiliated with Guangxi Medical University to examine GTSE1 expression in LUAD tissues and its regulatory mechanisms. The results demonstrated that GTSE1 was upregulated in LUAD tissues and had the potential to distinguish LUAD, consistent with previous research. However, when investigating the role of highly expressed GTSE1 in LUAD development, conflicting interpretations among researchers were discovered. Tian et al. (13) analyzed 246 samples from four public datasets obtained from the GEO database (GSE7339, GSE3268, GSE7670, GSE10072) and found that GTSE1 elevation had no significant association with clinical features and overall survival rates. In contrast, Kaushik et al. (16) analyzed 190 samples from four public datasets obtained from the GEO database (GSE19188, GSE19804, GSE101929, and GSE18842) and concluded that GTSE1 was upregulated in LC tissue, with differential expression associated with tumor venous invasion, patient survival time, and tumor maximum diameter. To investigate the relationship between high GTSE1 expression and prognosis in LUAD patients, we analyzed the clinical characteristics and survival time of the included samples. Our results revealed that upregulation of GTSE1 led to shorter survival time in LUAD patients, and high GTSE1 expression had a more accurate predictive effect on patient survival time. Although we had to eliminate some datasets during the analysis due to missing data and heterogeneity, it’s worth noting that the sample size on which these conclusions were based exceeded existing research, rendering our conclusions relatively more reliable at present.

Initially, GTSE1 was reported to negatively regulate p53 protein levels and p53-dependent cell apoptosis, thus promoting malignant tumor development (17). Further research has revealed potential mechanisms by which overexpressed GTSE1 is involved in regulating LC. In this study, through enrichment analysis, we found that upregulated GTSE1 is likely to promote LUAD development by participating in the cell cycle, p53 signaling pathway, and ubiquitin-mediated protein degradation, leading to a worsened prognosis for patients. Zheng et al. (18) suggest that knocking down GTSE1 leads to dysregulation of mitotic S phase in tumor cells, disrupting the cell cycle and promoting tumor cell apoptosis, which may explain the better prognosis in patients with low GTSE1 expression. In various malignancies, GTSE1 has been reported to be involved in p53-dependent cellular apoptosis, and downregulation of GTSE1 expression renders tumor cells more sensitive to chemotherapy agents (7, 19, 20). Notably, no studies have been found regarding the involvement of GTSE1 in tumor development regulation through ubiquitin-mediated protein degradation. Additionally, while investigating the relationship between GTSE1 and immune infiltration in LUAD cells, some studies have examined the relationship between GTSE1 and immune-related cells and functions in other tumor cells. Lei et al. (21) found that high GTSE1 expression in renal cell carcinoma was associated with increased levels of immune cell infiltration, leading to a worse prognosis for patients. Although our research conclusions align with existing studies, the aforementioned research was conducted on other malignancies. We might be the first to propose that GTSE1 potentially promotes LUAD development through involvement in the cell cycle, p53 signaling pathway, and ubiquitin-mediated protein degradation. Increased immune cell infiltration may be one of the contributing factors to the poor prognosis associated with high GTSE1 expression.

In this study, we observed that LUAD cells with low GTSE1 expression were sensitive to paclitaxel, cisplatin, gemcitabine, and pemetrexed, whereas LUAD cells with high GTSE1 expression demonstrated sensitivity to erlotinib, a targeted drug. Interestingly, our results indicated that first- and second-line chemotherapy drugs were primarily effective in LUAD cells with low GTSE1 expression, while targeted drugs showed efficacy in LUAD cells with high GTSE1 expression. Liu et al. suggested that knockout of the long non-coding RNA AK001796 indirectly inhibits GTSE1 expression, thereby improving sensitivity to cisplatin in LC (22). Xie et al. (20) found that high GTSE1 expression in osteosarcoma tissues reduced sensitivity to cisplatin, as it accelerated the S/G2 phase transition and enhanced DNA replication. Bublik et al. (23) also proposed that GTSE1 enhances the stability of p21 (CIP1/WAF1), counteracting the cytotoxicity induced by paclitaxel. However, there are no existing reports on the association between high GTSE1 expression in LUAD and sensitivity to targeted drugs. Due to limited research on GTSE1, we included literature from other cancer types. Nonetheless, our viewpoint is worth acknowledging: the sensitivity of LUAD cells with low GTSE1 expression to chemotherapy drugs is not coincidental, and downregulation of GTSE1 may potentially regulate the sensitivity of LUAD cells to chemotherapy drugs through specific biological pathways. Future studies should investigate the sensitivity of LUAD cells with high GTSE1 expression to targeted drugs and explore new therapeutic strategies, such as combining targeted drugs with chemotherapy drugs or developing novel GTSE1 inhibitors.

This study had several limitations. Firstly, the majority of samples were obtained from public databases, with a limited number of samples from the First Affiliated Hospital of Guangxi Medical University. Hence, additional experiments are necessary to validate the expression levels and clinical significance of GTSE1 in LUAD tissues. Secondly, the relationship between GTSE1 expression and the tumor immune microenvironment, as well as the signaling pathways involving GTSE1 in regulating LUAD development, remains unclear, necessitating further investigation. Thirdly, while GO and GSEA analyses provided insights into the potential pathways GTSE1 may be involved in, these results alone are not sufficient to fully determine GTSE1’s function and role in LUAD. Further experimental validation and mechanistic studies are needed to understand how GTSE1 influences LUAD through these pathways. Lastly, more research is needed to explore the relationship between GTSE1 expression levels and the effectiveness of chemotherapy or targeted therapy drugs in LUAD.

## Conclusion

In summary, GTSE1 demonstrates potential as a valuable biomarker for diagnosing LUAD, predicting unfavorable outcomes, and guiding medication selection. Its expression level correlates with immune cell infiltration in the tumor and may contribute to immune evasion, providing new insights for the clinical management of LUAD. We propose that GTSE1 could potentially promote LUAD development through its involvement in the cell cycle, p53 signaling pathway, and ubiquitin-mediated protein degradation. Future research will delve into the functional aspects and underlying mechanisms of GTSE1.

## Data Availability

The original contributions presented in the study are included in the article/Supplementary material, further inquiries can be directed to the corresponding author.
