# Supplementary material for: Comprehensive analysis of the diagnostic and therapeutic value, immune infiltration, and drug treatment mechanisms of GTSE1 in lung adenocarcinoma
Source: Front Med (Lausanne). 2024 Nov 19;11:1433601. doi: 10.3389/fmed.2024.1433601 (PMC11611587; doi:10.3389/fmed.2024.1433601)
Supplement: Supplementary file 1 [file Data_Sheet_1.docx]

**Supplementary Information**


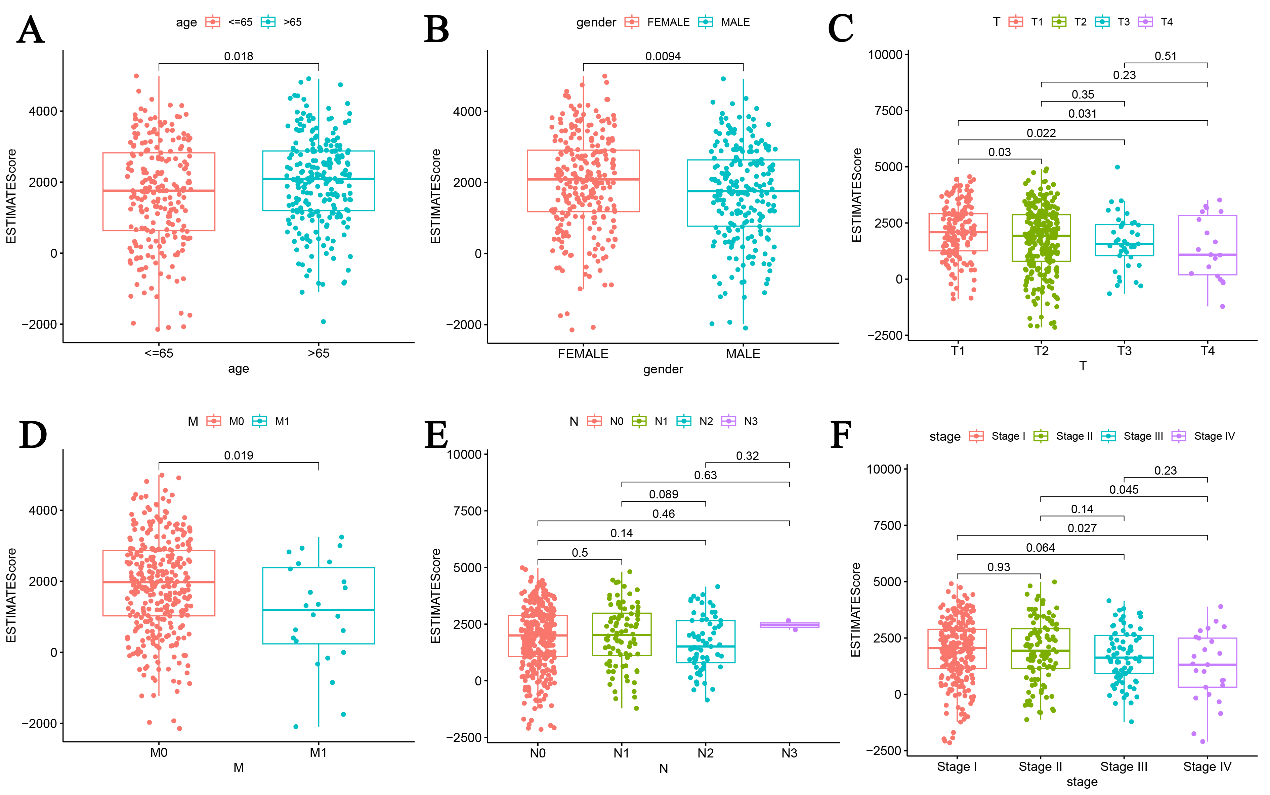


Additional file 1: Fig. S1. The connection between GTSE1 expression and clinicopathological parameters. (A) Age, (B) Gender, (C) T stage, (D) M stage, (E) N stage, (F) AJCC stage.
