# Supplementary material for: Comprehensive analysis of the diagnostic and therapeutic value, immune infiltration, and drug treatment mechanisms of GTSE1 in lung adenocarcinoma
Source: Front Med (Lausanne). 2024 Nov 19;11:1433601. doi: 10.3389/fmed.2024.1433601 (PMC11611587; doi:10.3389/fmed.2024.1433601)
Supplement: Supplementary file 2 [file Data_Sheet_2.docx]

**Supplementary Information**


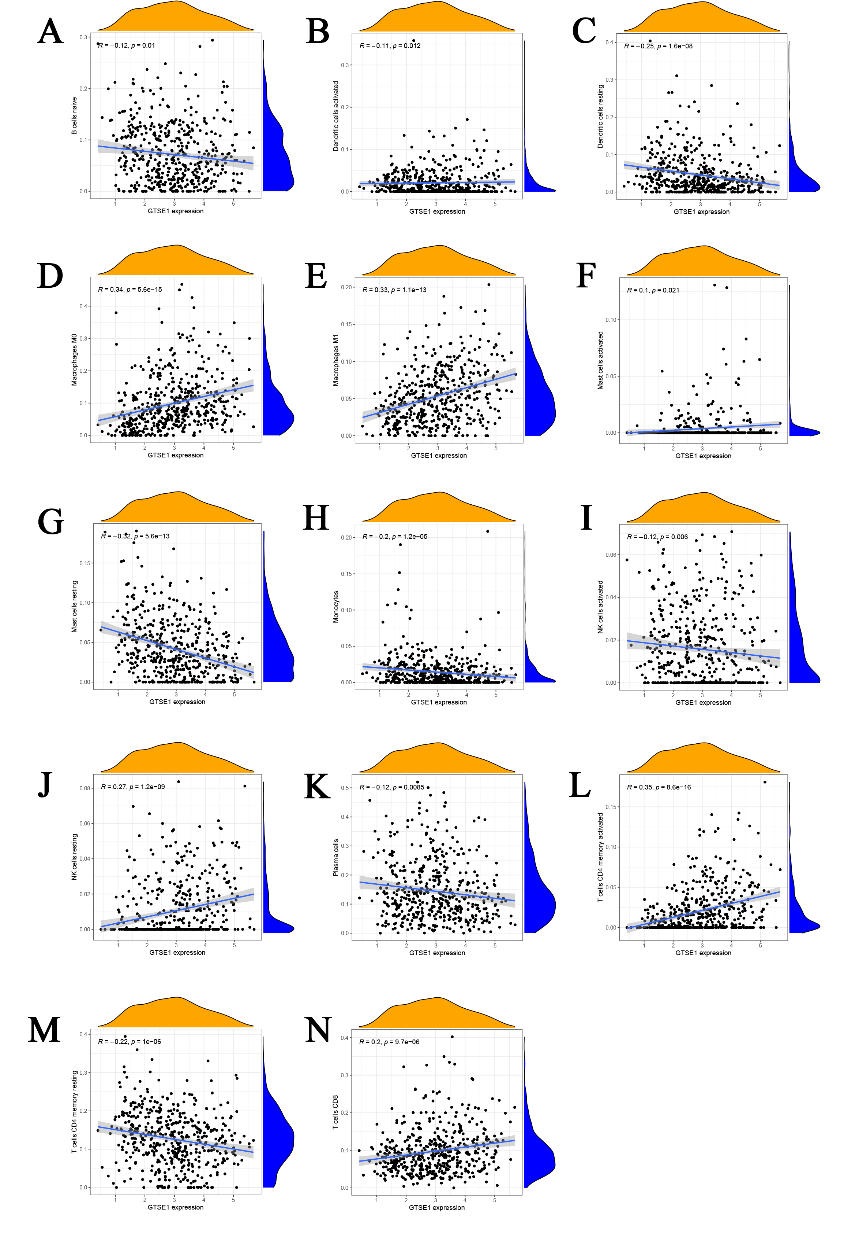


Additional file 2: Fig. S2. (A-N) The relationship between GTSE1 expression and fourteen immune infiltrates in lung adenocarcinoma (LUAD) from the Tumor Immune Estimation Resource (TIMER).
