# Supplementary material for: Comprehensive analysis of the diagnostic and therapeutic value, immune infiltration, and drug treatment mechanisms of GTSE1 in lung adenocarcinoma
Source: Front Med (Lausanne). 2024 Nov 19;11:1433601. doi: 10.3389/fmed.2024.1433601 (PMC11611587; doi:10.3389/fmed.2024.1433601)
Supplement: Supplementary file 3 [file Data_Sheet_3.docx]

**Supplementary Information**


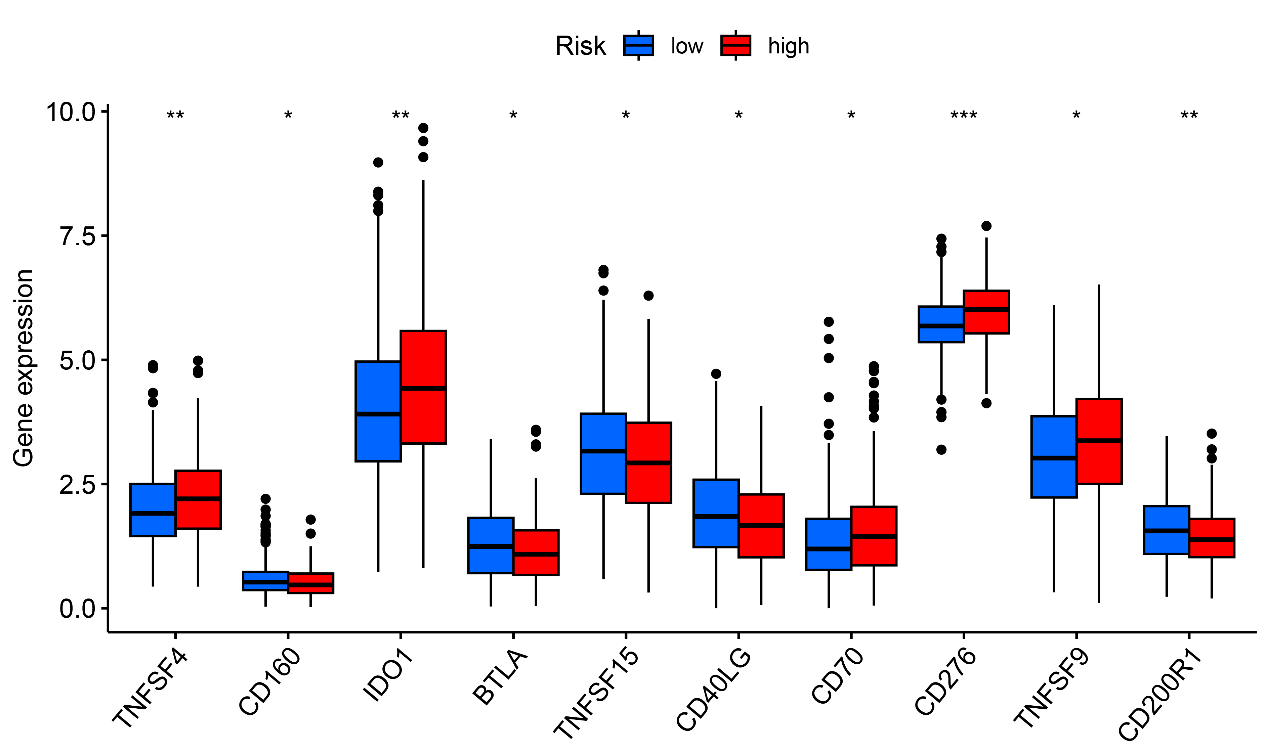


Additional file 3: Fig. S3. Differential expression of immune checkpoint related genes between high and low GTSE1 expression groups. (*, p<0.05; **, p<0.01; ***, p<0.001).
